# Key Edaphic Properties Largely Explain Temporal and Geographic Variation in Soil Microbial Communities across Four Biomes

**DOI:** 10.1371/journal.pone.0135352

**Published:** 2015-11-04

**Authors:** Kathryn M. Docherty, Hannah M. Borton, Noelle Espinosa, Martha Gebhardt, Juliana Gil-Loaiza, Jessica L. M. Gutknecht, Patrick W. Maes, Brendon M. Mott, John Jacob Parnell, Gayle Purdy, Pedro A. P. Rodrigues, Lee F. Stanish, Olivia N. Walser, Rachel E. Gallery

**Affiliations:** 1 Department of Biological Sciences, Western Michigan University, Kalamazoo, Michigan, United States of America; 2 School of Natural Resources and the Environment, University of Arizona, Tucson, Arizona, United States of America; 3 Department of Soil, Water and Environmental Science, University of Arizona, Tucson, Arizona, United States of America; 4 Department of Soil, Water, and Climate, University of Minnesota, Twin Cities, St. Paul, Minnesota, United States of America; 5 Graduate Interdisciplinary Program in Entomology and Insect Science, University of Arizona, Tucson, Arizona, United States of America; 6 USDA-ARS Carl Hayden Bee Research Center, Tucson, Arizona, United States of America; 7 National Ecological Observatory Network, Boulder, Colorado, United States of America; 8 Department of Ecology and Evolutionary Biology, University of Arizona, Tucson, Arizona, United States of America; U. S. Salinity Lab, UNITED STATES

## Abstract

Soil microbial communities play a critical role in nutrient transformation and storage in all ecosystems. Quantifying the seasonal and long-term temporal extent of genetic and functional variation of soil microorganisms in response to biotic and abiotic changes within and across ecosystems will inform our understanding of the effect of climate change on these processes. We examined spatial and seasonal variation in microbial communities based on 16S rRNA gene sequencing and phospholipid fatty acid (PLFA) composition across four biomes: a tropical broadleaf forest (Hawaii), taiga (Alaska), semiarid grassland-shrubland (Utah), and a subtropical coniferous forest (Florida). In this study, we used a team-based instructional approach leveraging the iPlant Collaborative to examine publicly available National Ecological Observatory Network (NEON) 16S gene and PLFA measurements that quantify microbial diversity, composition, and growth. Both profiling techniques revealed that microbial communities grouped strongly by ecosystem and were predominately influenced by three edaphic factors: pH, soil water content, and cation exchange capacity. Temporal variability of microbial communities differed by profiling technique; 16S-based community measurements showed significant temporal variability only in the subtropical coniferous forest communities, specifically through changes within subgroups of *Acidobacteria*. Conversely, PLFA-based community measurements showed seasonal shifts in taiga and tropical broadleaf forest systems. These differences may be due to the premise that 16S-based measurements are predominantly influenced by large shifts in the abiotic soil environment, while PLFA-based analyses reflect the metabolically active fraction of the microbial community, which is more sensitive to local disturbances and biotic interactions. To address the technical issue of the response of soil microbial communities to sample storage temperature, we compared 16S-based community structure in soils stored at -80°C and -20°C and found no significant differences in community composition based on storage temperature. Free, open access datasets and data sharing platforms are powerful tools for integrating research and teaching in undergraduate and graduate student classrooms. They are a valuable resource for fostering interdisciplinary collaborations, testing ecological theory, model development and validation, and generating novel hypotheses. Training in data analysis and interpretation of large datasets in university classrooms through project-based learning improves the learning experience for students and enables their use of these significant resources throughout their careers.

## Introduction

Through their predominant roles in carbon (C) and nitrogen (N) cycling, and their positive and negative feedbacks with plant communities, soil microorganisms drive and influence the outcome of ecosystem function, services, and successful conservation and restoration strategies. Physical and chemical properties of soil provide niche space for biological inhabitants and influence community resilience to local disturbances and regional climatic shifts [1]. For example, understanding soil microbial resilience and adaptation to environmental change is especially important considering their role in the stability of soil C storage and in driving rates of greenhouse gas (CO_2_ or CH_4_) release to the atmosphere [2–5], with critical implications for accurately predicting global change parameters. Specific assemblages of microbes can differentially influence rates of litter decomposition and nitrogen mineralization [6], and the results of numerous soil warming experiments reveal the importance of microbial community structure and physiological potential for acclimatization as drivers of C turnover and storage [7–10]. The resilience and adaptation of soil microbial communities to complex environmental change thus remains an active area of research.

16S rRNA-based sequencing and metagenomic surveys have been effectively used to conduct spatial characterization of soil bacterial communities across environmental gradients [11–14]. Overwhelmingly, 16S-based bacterial diversity in soil is far greater than in most other environments [15], and differences in composition of major bacterial phyla are best explained by edaphic properties such as pH, soil moisture, and texture [12], [16]. Thus, current evidence strongly suggests that controls of microbial community distributions differ from those observed for plants and animals at regional and global scales [17], and highlights the important need for studies addressing dispersal limitation and specific environmental filters to specific microbial functional groups and taxa.

Soil bacterial communities characterized by the16S rRNA gene are also temporally stable compared to other microbial habitats [18], but land use history can impact how seasonal and other temporal variation influences soil microbial diversity [16], [19]. Though soil microbial communities might be relatively stable, the population turnover of individual microbial taxa is dynamic and driven by species-specific responses to common environmental drivers. For example, alpine dry meadow bacterial communities show strong seasonal changes in both diversity and relative abundance within taxa in response to snowmelt [20]. Furthermore, comparisons of microbial community structure using DNA-based and RNA-based approaches show that not all taxa present in a microbial community are active at a given point [21], [22]. In fact, accumulating evidence shows that at any snapshot in time, a significant proportion of the microorganisms in soils may be dormant or senescent [23], [24]. More studies are needed to identify conditions where analyses of microbial composition (i.e., DNA-based) and microbial growth and activity (i.e., lipid-based and RNA-based) align, and, perhaps more importantly, the circumstances under which they do not align. Additionally, while extremely powerful, 16S-based studies may limit our understanding of soil function because they examine bacterial communities in isolation from other important soil organisms, such as fungi, which can directly and indirectly impact bacterial community structure [25]. Knowledge of the proportion of the community that is active and responding to environmental drivers being measured is needed to make predictions regarding their temporal stability and resilience to pulse disturbance events, seasonal shifts in energy and water availability, and longer term environmental change.

This study is the result of a multidisciplinary, project-based course using open-access publicly available data and cyber-infrastructure. This undergraduate-graduate course taught concurrently via videoconferencing at Western Michigan University (Kalamazoo, MI) and the University of Arizona (Tucson, AZ) focused on exploring the spatial and temporal layers and drivers of microbial community composition within and across four terrestrial ecosystems over an annual cycle. With the knowledge that interdisciplinary collaborations foster better science by uniting different expertise to include a variety of perspectives, modes of formulating questions, and research approaches [26], [27], this course used a team-based framework [28] to develop and establish priority research questions, integrate background literature, and analyze and synthesize microbial 16S rRNA gene sequence, phospholipid fatty acid, fatty acid methyl ester, and biogeochemical datasets. Course participants used publicly available data from the National Ecological Observatory Network (NEON; neoninc.org), the data sharing and interactive infrastructure of the iPlant Collaborative (iplantcollaborative.org), and popular online collaboration tools to share ideas and educational resources that facilitated peer discussion and mentoring. Written and video resources created by course participants for data analysis are located here: https://sites.google.com/site/dochertyetal2015plosone/.

## Methods

### Soil Sampling

**Fig 1:**
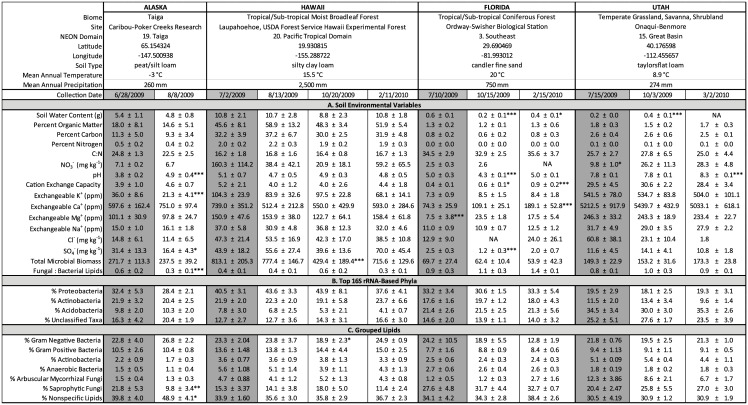
Average values for all measured (A) soil environmental variables, (B) dominant 16S rRNA-determined bacterial phyla, and (C) grouped lipids ± 95% confidence intervals for all soil samples at all time points. Columns in gray indicate time points closest to peak greenness at each site, which are used for cross-site comparisons. Significant differences over time, within sites, are indicated with * (p < 0.05), ** (p < 0.01), *** (p < 0.001), as compared to the time point at peak greenness using repeated measures ANOVA. Differences between sites are not indicated here, but are described in Fig 2A.

From 2009–2010, 408 soil samples were collected and a subset were analyzed from four biomes that represent a broad latitudinal gradient with unique soil properties and distinct climates with different levels of intra-annual variability in temperature and precipitation, as defined by the NEON design (Fig 1). Soils were collected from taiga (NEON Domain 19; Caribou-Poker Creek, Alaska), tropical/subtropical moist broadleaf forest (NEON Domain 20; Laupahoehoe, Hawaii), tropical/sub-tropical coniferous forest (NEON Domain 3; Ordway-Swisher Biological Station, Florida) and temperate grassland/savanna/shrubland (NEON Domain 15; Onaqui-Benmore, Utah). Sampling did not require permits and did not involve endangered or protected species. For more NEON site information see http://www.neoninc.org/science-design/field-sites. Sampling dates were designated to include the beginning and end of growing seasons, as well as periods representative of annual temperature or precipitation extremes. Samples for microbial community analysis were collected within a grid measuring 160 × 320 m divided into eight 80 × 80 m cells. Soil cores (7.5 cm in diameter encompassing the 0–10 cm depth interval below the litter layer) were collected from three randomly assigned GPS coordinates within each of the eight cells. In addition, sub-sets of the three cores corresponding to each cell were combined for a composite sample representative of each cell. Cores within each grid were homogenized, sieved through 2 mm mesh, and subsamples were either air-dried or frozen at -80°C for specific downstream analyses. Subsamples of sieved soils collected from Hawaii were frozen at both -20°C and -80°C to compare the effect of storage temperature on microbial community composition.

### Environmental Metadata and Soil Chemistry Measurements

A suite of abiotic and site characteristics following Minimum Information about any (X) Sequence (MIxS) descriptions developed by the Genomics Standards Consortium [29] corresponds with each microbial sample. Soil biogeochemistry assays (Fig 1A) were performed at the University of Wisconsin Soil Testing Laboratories, and specific protocol for each assay, including pH, soil water content (SWC), and Cation Exchange Capacity (CEC) can be found at http://uwlab.soils.wisc.edu/lab-procedures/. For Alaska, Florida, and Utah, peak greenness dates were estimated using a 2001–2009 average from the MODIS Terra Vegetation Indices enhanced vegetation index (EVI) product [30].

### Lipid Analysis

We used a hybrid procedure combining phospholipid fatty acid (PLFA) and fatty acid methyl ester (FAME) analysis to analyze microbial community composition [31], [32]. The procedure was based on the extraction of ‘signature’ lipid biomarkers from the microbial cell membrane. Membrane lipids were extracted, purified, and identified using steps from a modified [33] technique for lipid extraction, combined with FAME analysis as described by Microbial ID Inc. (Hayward, CA, USA). Lipids were extracted from 3 g of freeze-dried soil three times using a chloroform extraction (4 ml) with phosphate buffer (potassium phosphate (3.6 ml) and methanol (8 ml). The phases were then allowed to separate overnight at room temperature, followed by reduction of the chloroform phase volume (after aspiration to remove the aqueous phase) in a RapidVap evaporator. The procedure for FAME was then conducted (Microbial ID Inc, Hayward CA, USA); saponification followed by strong acid methanolysis and phase separation to extract the methyl-esterfied fatty acids. A 2 μl injection of the methyl-esterfied fatty acids was analyzed using a Hewlett-Packard 6890 Gas Chromatograph equipped with a flame ionization detector and an Ultra 2 capillary column (25 ml, 0.2 mm diameter, 0.33 μm film thickness; 5%-phenyl, 95% methyl) (Agilent Technologies Inc., Santa Clara, CA, USA). Lipid peaks were identified using bacterial fatty acid standards and MIDI peak identification software (“Sherlock microbial identification system”, MIDI Inc, Newark, DE, USA). Peak areas were converted to nmol lipid g dry soil^-1^ using internal standards (9:0 nonanoic methyl ester and 19:0 nonadecanoic methyl ester). The total nmol lipid g dry soil^-1^ (sum of all lipids present, 20 or less carbons in length) was used as an index of microbial biomass [34–37]. Individual lipids were used as biomarkers to indicate broad groups within the microbial community: 16: 1 ωc for arbuscular mycorrhizal fungi (AMF) [31]; an average of 18:1 ω9c and 18: 2 ω6,9c for general fungi excluding AMF (general fungal, (GF) [31]; an average of monounsaturated and hydroxy fatty acids for 16: 1 x7c for Gram-negative bacteria [38]; an average of cyclopropyl fatty acids for anaerobic bacteria [34], and an average of methyl side-chained fatty acids and 15: 0 iso for Gram-positive bacteria [38]. The ratio of fungal lipids (average 16: 1 xc5, 18: 1 x9c, and 18: 2 x6,9c) to bacterial lipids (average 15: 0 iso, 15: 0 anteiso, 16: 0 2OH, 16: 0 iso, 16: 1 x7c, 16: 0 10 methyl, 17: 0 iso, 17: 0 anteiso, 17: 0 cyclo, 18: 1 x5c, and 18: 1 x7c) was used to indicate the fungal to bacterial ratio [37].

### DNA Extraction and 16S rRNA Analysis

DNA from 0.25 g of each of the soil samples was extracted using the MoBio PowerSoil DNA isolation kit (MoBio Laboratories, Carlsbad, CA) following procedures described in [11]. Universal primers (515f/806r) [39] were used to amplify the V3-V4 region of the 16S rRNA gene, which provides optimal community clustering to examine the richness and community composition of bacteria and archaea [40–42]. Pooled triplicate PCR amplifications were sequenced at Engencore (University of South Carolina) using Roche 454 GS-FLX pyrosequencing with titanium chemistry. Sequences were aligned using the Greengenes core reference alignment [43] and raw reads from the .sff file provided from Engencore were processed using mothur v.1.33.3 [44]. Unless otherwise stated, each command used the default parameters. The sequences were quality filtered using the trim.flows command (flows = 450) and those with more than 2 base mismatches to the 806R primer sequence or 1 mismatch to the 10 base pair identification barcode were removed. All reads <200 base pairs were removed using the trim.seqs command. The remaining sequences were aligned to Silva SSURef database (v102) using the align.seqs command. Sequences that started before the 806R sequence or that were shorter than 98% of the sequences were eliminated using the screen.seqs command. Sequences that were within 1% similarity were clustered together using the pre.cluster command. Chimeras were removed using a *de novo* check in uchime [45] and any sequences that were of mitochondrial, chloroplast, Archaeal, or Eukaryote origin were removed using the remove.seqs command. A distance matrix was constructed for the aligned sequences using the dist.seqs command. Using the make.shared command, the sequences were binned into operational taxonomic units (OTUs) based on 97% sequence similarity. Representative sequences from each OTU were classified with the RDP Naïve Bayesian Classifier (version 9 training set [46]. All sequences, mapping files, and metadata files that include PLFA data (see also S1 Table) generated in this study are freely available via the iPlant Collaborative and are publicly available to download. A user account is free and data can be accessed at: https://de.iplantcollaborative.org/de/?type=data&folder=/iplant/home/shared/NEON_Pilot. Raw sequence data in FASTQ format are accessible from the NCBI SRA study number SRP061236, accession numbers SRX1098949-SRX1292693.

### Statistical Analysis

To standardize ecosystem-level comparisons, we determined the time point sampled closest to the highest net primary productivity (i.e., Enhanced Vegetation Index [EVI] peak greenness) at each site using MODIS data [30], and used this time point to compare environmental and microbial community variables among the four ecosystems. Cross-site comparisons of 16S rRNA- and lipid-based soil microbial community composition were conducted at the date most closely corresponding to peak greenness. Within site, seasonal changes in microbial community composition were compared. For the 16S-based analyses, we rarefied to 1000 OTUs per soil sample and excluded samples containing less than 1000 OTUs and then calculated the relative abundance of each identified phylum and all unclassified taxa. For the lipid-based analyses, we calculated the relative abundance of each unique lipid determined by GC analysis in each soil sample. At peak greenness, relative abundance data for each type of microbial community profiling technique were visualized using a nonmetric multidimensional scaling (NMDS) approach using a Bray-Curtis calculation for the dissimilarity matrix. We determined whether sample location (site) or the time of sampling (date) had an effect on microbial DNA or lipid based community composition using analysis of similarity (ANOSIM), with a Bray-Curtis dissimilarity measure. We also examined the overlap of communities using analysis of variance (ANOVA) and pairwise Tukey tests on scores from NMDS analyses (JMP software v. 10). We normalized all measured soil environmental variables (Fig 1) using individualized power transformations and tested for the assumption of normality using a Shapiro-Wilk test. Following normalization, we performed an ANOVA to determine differences in environmental factors by site at peak greenness, as well as with the grouped lipids (Fig 1) and all 16S-based phyla and unclassified sequences (Fig 1). Then we performed permutational multivariate analysis of variance (permanova) to determine which environmental variables best explained 16S rRNA gene- and lipid-based community dissimilarity. Again, we used a Bray-Curtis dissimilarity measure for permanova analysis. Finally, we used stepwise multiple linear regression analysis to examine which environmental factors had the greatest influence in variation of MDS1 (the x axis) and MDS2 (the y axis) of each NMDS plot.

We combined data from all dates, visualized data using NMDS, and tested for the effect of site and time among all sites with both microbial community-profiling methods. Again, we performed ANOSIM and permanova analyses to test for the effect of site, time, and the environmental variables listed in Fig 1. We repeated these analyses using time-series community data from each individual site. When the effect of time was significant, we further examined variation at the Order level for 16S-based data from the five most abundant phyla and with categorized lipids for the lipid-based data.

We used 16S data collected from Hawaiian soils only to examine the effect of storage temperature (-20°C vs. -80°C) on microbial community structure. We visualized 16S-based communities using NMDS and performed ANOSIM to determine the effect of temperature on community composition. All statistical analyses were performed in the R (version 3.0.1) statistical environment using R studio (version 0.97.551). We used the vegan package to perform all multivariate analyses [47].

## Results

### Differences Among Sites at Peak Greenness

**Fig 2:**
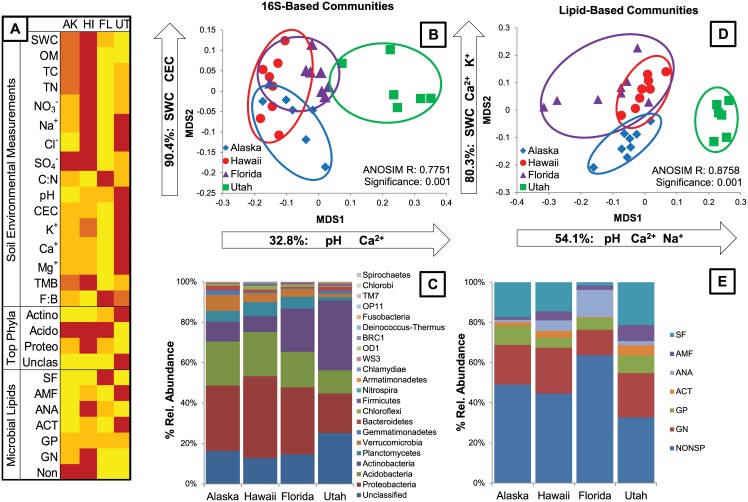
Microbial community variation across sites at the time point closest to peak greenness for the site. (A) Heatmap representing significant differences (α = 0.05) between soil environmental variables, the most abundant 16S-based phyla and grouped lipids. Changes in color represent a significant difference, where red is the highest average value and yellow is the average value. (B) NMDS ordination of 16S rRNA-based communities at the phylum level indicates significant differences in community composition by site (ANOSIM R = 0.7751, significance = 0.001). pH and Ca^2+^ explaining 32.8% of the variation in MDS1; SWC and CEC explain 90.4% of the variation in MDS2. (C) Percent relative abundance of all unclassified taxa and identified phyla using 16S rRNA data. (D) NMDS ordination of lipid-based communities indicates significant differences in community composition by site (ANOSIM R = 0.8758, significance = 0.001). pH, Ca^2+^ and Na^+^ explain 54.1% of the variation in MDS1; SWC, Ca^2+^ and K^+^ explain 80.3% of the variation in MDS2. (E) Relative abundance of nonspecific and diagnostic lipids for Gram Negative Bacteria (GN), Gram Positive Bacteria (GP), Actinobacteria (ACT), Anaerobic Bacteria (ANA), Arbuscular Mycorrhizal Fungi (AMF) and Saprophytic Fungi (SF).

We determined the time point sampled closest to the peak greenness at each site using MODIS data and used these time points to characterize environmental and microbial community variables (16S rRNA gene and PLFA) among the four ecosystems. Overall, soil chemistry and microbial communities differed significantly between the sites. In general, the sites can be divided based on two groups of environmental variables (Fig 2A). The soils from sites in taiga (Alaska) and tropical moist broadleaf forest (Hawaii) biomes contained higher soil water content (SWC), organic matter (OM), total carbon (TC), total nitrogen (TN) and sulfate (SO_4_^−^) than soils collected in sub-tropical coniferous forest (Florida) and temperate grassland/savanna/shrubland (Utah). Soils from Utah were distinctive based on higher pH, cation exchange capacity (CEC), and cation concentrations (K^+^, Ca^+^, Mg^+^) than soils from the other three sites (Figs 1 and 2A). Conversely, soils collected from the Florida site contained the lowest cation concentrations and lowest CEC, distinguishing them from the Utah soils (Figs 1 and 2A). Total microbial biomass (TMB) differed significantly between each of the four sites and was highest in soils from Hawaii, then Alaska, then Utah, and lowest in Florida soils (Figs 1 and 2A). Shifts in microbial biomass were inversely related to the fungal:bacterial ratios of the four soils, such that Florida soils contained the highest fungal:bacterial ratio of lipids while Hawaiian soils contained the lowest ratio (Figs 1 and 2A).

We examined the soil microbial communities at peak greenness by comparing 16S rRNA sequences obtained via a high throughput pyrosequencing from eight replicate soil cores at each site. One replicate from the Alaska site and two replicates from the Utah site were lost during processing. The 16S-based communities differed significantly by site (ANOSIM R = 0.7751, significance = 0.001, Fig 2B). In general soil communities were separated by MDS1. Soil microbial community profiles from Utah differed completely from soils collected in Alaska, Hawaii, and Florida (p < 0.05, Table 1, Fig 2B). Microbial communities from Florida soils were also distinctive, but overlapped with communities in Alaskan soils on MDS1, and with both Alaska and Hawaii soils on MDS2 (Table 1, Fig 2B).

**Table 1:** ANOVA and pairwise Tukey’s analysis to determine differences between sites at peak greenness, based on PLFA and 16S analysis (corresponds to Fig 2).

	16S				PLFA			
Site	MDS1	SD1	MDS2	SD2	MDS1	SD1	MDS2	SD2
Alaska	-0.066	0.069^**bc**^	-0.074	0.065^**b**^	-0.057	0.051^**bc**^	-0.121	0.054^**c**^
Hawaii	-0.144	0.026^**c**^	0.010	0.076^**ab**^	-0.003	0.040^**b**^	0.070	0.057^**a**^
Florida	-0.021	0.058^**b**^	0.035	0.040^**a**^	-0.119	0.119^**c**^	0.074	0.069^**a**^
Utah	0.242	0.097^**a**^	0.014	0.057^**ab**^	0.239	0.018^**a**^	-0.031	0.068^**b**^
P-value	<0.0001		0.0199		<0.0001		<0.0001	

Superscript letter values indicate statistical differences between clusters of communities associated with MDS1 (x-axis) and MDS2 (y-axis). SD indicates standard deviation.

The measured environmental variables that best explained the differences in 16S-based community structure were soil pH (R^2^ = 0.30809, p = 0.001), SWC (R^2^ = 0.27591, p = 0.001) and CEC (R^2^ = 0.11292, p = 0.006). All other variables listed as edaphic properties in Figs 1 and 2A were included in the permanova, but did not explain a significant portion of 16S-based community dissimilarity. We excluded two environmental variables from the analysis (Cl^−^ and NO_3_^−^ concentrations) because there were too few successful replicates of these values. Using stepwise multiple linear regression analysis, we examined which environmental variables explained the variation of each MDS axis. SWC and CEC explained a combined 90.4% (F_2,25_ = 118.041, p < 0.001) of the variation in MDS2, while pH and Ca^2+^ concentration explained a combined 32.8% (F_2,25_ = 3.936, p = 0.03) of variation in MDS1 (Fig 2A).

The four sites were dominated by the same Bacterial phyla: *Proteobacteria*, *Acidobacteria*, *Actinobacteria*, *Planctomycetes*, and *Verrucomicrobia* (Fig 2C). Additionally, all communities contained a significant proportion of unclassified 16S rRNA sequences ranging from 13% in Hawaii soils to a maximum of 25% in Utah soils (Fig 2A). We visualized the communities using only the sequences representing unclassified taxa, *Proteobacteria*, *Acidobacteria*, and *Actinobacteria* using NMDS (not shown), and the same pattern of community similarity was obtained as shown in Fig 2B, indicating that variations in these top four groups can be used to explain broad community differences among sites. Taxa classified as *Proteobacteria* comprised the highest proportion of the total community in soils from Hawaii, but were lowest in soils from Utah, and equivalent in Alaska and Florida (Fig 2A and 2C). Taxa classified as *Acidobacteria* represented equivalent proportions of the total community in soils from Alaska, Hawaii and Florida, but were lower in Utah (Fig 2A and 2C). Taxa classified as *Actinobacteria* comprised the greatest proportion of the communities from Utah soils, were an intermediate proportion of Florida soil communities, and were lowest in soils from Alaska and Hawaii (Fig 2A and 2C). The shift in the ratio of *Proteobacteria* + *Acidobacteria* to *Actinobacteria* is the main factor driving the dissimilarity of Utah soils from the other three sites (Fig 2B and 2C).

In addition to examining soil communities using a 16S-based genetic approach, we also examined communities using a functional lipid-based approach. As with the 16S-based analysis, lipid profiles from eight replicate soils collected from each site at peak greenness were examined. Two replicate samples from the Utah site were lost during processing. As with the 16S-based communities, lipid-based communities differed significantly by site (ANOSIM R = 0.8758, p = 0.001, Fig 2D) and differed significantly on both NMDS axes (p < 0.001). Lipid-based soil community composition in the Utah soils differed significantly from the composition of communities in the Hawaii, Alaska and Florida soils (p < 0.05 on MDS1 and MDS2 Table 1, Fig 2D). Alaska soils were also distinct on MDS2, but had some overlap with Hawaii and Florida soils on MDS1 (Table 1, Fig 2D). Community composition in Florida soils varied more among the four sites, while replicates from Alaska, Hawaii and Utah soils varied less (Table 1), indicating lower spatial variability at these sites. Florida and Hawaii soils contained the highest amount of overlapping lipid composition, but soils from Alaska, Hawaii, and Florida still differed significantly in composition either on MDS1 or MDS2 (p < 0.0001 for overall ANOVA to test site effects, Table 1, Fig 2D).

As with the 16S-based communities, the environmental variables that best explained the dissimilarity in lipid-based community composition were soil pH (R^2^ = 0.14715, p = 0.001), SWC (R^2^ = 0.15539, p = 0.001) and CEC (R^2^ = 0.18690, p = 0.001). Additionally, Ca^2+^ ion concentrations (R^2^ = 0.06469, p = 0.004) and soil C:N (R^2^ = 0.07477, p = 0.003) explained significant amounts of the variation in lipid-based community composition between sites. All other variables listed as soil environmental measurements in Figs 1 and 2A were included in the permanova, but did not explain a significant portion of community dissimilarity. Again, we excluded Cl^−^ and NO_3_^−^ concentrations from this analysis. We also excluded TMB and the fungal:bacterial ratio because they are not independent from lipid data. Using stepwise multiple linear regression analysis, we examined which environmental variables explained the variation of each NMDS axis. SWC, Ca^2+^ and K^+^ ion concentrations explained a combined 80.3% (F_3,24_ = 32.672, p < 0.001) of the variation in MDS2, while pH, Ca^2+^ and Na^+^ ion concentrations explained a combined 54.1% of the variation in MDS1 (Fig 2D).

Examining among-site differences in lipid-based community composition, the four sites all contained higher proportions of nonspecific lipids than of any defined groups of lipids (Fig 2E). Nonspecific lipid proportions ranged from 33% in Utah soils to 64% in Florida soils. All soils contained equivalent amounts of lipids diagnostic for Gram Positive Bacteria (GP), but soils from Hawaii had a higher proportion of Gram Negative (GN) and Anaerobic (ANA) bacterial lipids than the other sites (Fig 2A and 2E). Utah soils contained the highest proportion of lipids that are diagnostic for *Actinobacteria* (ACT), reflecting results seen with 16S-based communities. Utah soils also contained the highest proportion of lipids diagnostic for arbuscular mycorrhizal fungi (AMF), while Alaska and Florida soils contained equally low amounts of AMF and Hawaiian soils were intermediate. Finally, the proportion of Saprophytic Fungi (SF) was highest in Florida soils, but equally lower in soils from the other three sites (Fig 2A and 2E).

We observed similar differences among soil microbial communities at the four sites using both 16S- and lipid-based approaches. Communities in Utah soils were most distinctive from the other three sites and communities from Hawaii and Florida tended to overlap more than any other two sites, regardless of the technique used to assess community composition (Fig 2B and 2D). Similar environmental factors explained significant proportions of variation in community composition. Soil pH, SWC, and CEC explained the majority of community dissimilarity using both methods. Further, SWC explained a large proportion of variation in MDS2 and pH explained a large proportion of variation in MDS1 using both methods, indicating that similar community profiles, controlled by the same environmental variables, were obtained by both 16S- and lipid-based approaches when comparing across sites at peak greenness.

### Temporal Differences Among Sites

We examined changes in soil environmental variables and microbial communities over time. At the Alaska site, soils from only two dates (June 28, 2009 and August 8, 2009) were included in the analyses. For the remaining sites, 3–4 dates spanning a longer time scale from July 2009 –March 2010 were included in the analyses. Fig 1 indicates the exact dates when soils were collected from each site, average values for all data, and significant differences over time. The measured environmental variables of soils collected from Alaska, Hawaii, and Utah exhibited little variation, while soils collected from Florida were variable over time. In soils collected from Alaska, soil pH increased significantly from June to August (F_1, 13_ = 20.877, p = 0.001). Additionally, soil K^+^ and SO_4_^−^ concentrations decreased significantly over time (F_1,13_ = 10.119, p = 0.007 for K^+^; F_1,13_ = 5.44, p = 0.036 for SO_4_^−^), and the fungal:bacterial ratio also decreased over time (F_1,13_ = 10.774, p = 0.006). At the Hawaiian site, soils exhibited a significant change in TMB over time due to a decrease in biomass in the October 2009 samples, as compared to the July 2009, August 2009 and February 2010 samples (F_3,23_ = 5.259, p = 0.007). Soils collected from Utah exhibited a significant increase in pH in March 2010 (F _2,15_ = 16.808, p < 0.001) as compared to samples collected in July and October 2009. Additionally, SWC in Utah soils increased from July to October 2009 (F_2,15_ = 91.719, p < 0.001), and soil NO_3_^−^ concentrations increased from July 2009 to October 2009 and March 2010 (F_2,11_ = 6.139, p = 0.016). The measured environmental variables in soils collected from Florida were more variable over time. Soil pH decreased in October 2009, compared to higher pH levels in July 2009 and February 2010 (F_2,20_ = 22.132, p < 0.001). SWC varied at each date (F_2,20_ = 31.123, p < 0.001) and CEC increased incrementally over the three sample dates (F_2,20_ = 12.722, p < 0.001). Ca^2+^ ion concentrations were significantly higher in February 2010 than the other two dates (F_2,20_ = 11.55, p < 0.001) and Mg^+^ ion concentrations were significantly lower in July 2009 than the other two dates (F_2,20_ = 534.969, p < 0.001). Finally, SO_4_^−^ concentrations were lower in October 2009 than in July 2009 or February 2010 (F_2,20_ = 6.654, p = 0.006, Fig 1).

**Fig 3:**
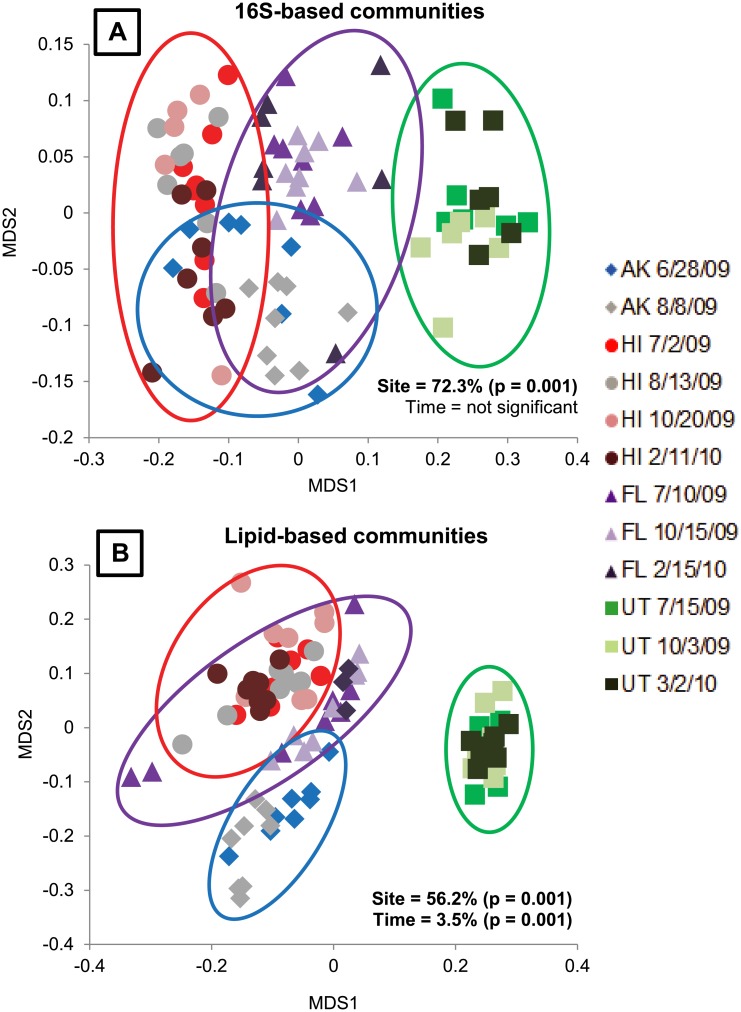
Microbial community variation across sites at all time points using NMDS ordination. A) Variation in 16S rRNA based communities was only significant by site (F = 69.003, p = 0.001) and not by time (F = 1.003, p = 0.332). The environmental variables that explained significant variation in 16S-based communities were pH, SWC, CEC, Na^+^, Mg^+^ and Ca^2+^. B) Variation in lipid-based communities was significant by site (F = 38.964, p = 0.001) and over time (F = 7.381, p = 0.001). The environmental variables that explained significant variation in the lipid-based communities were SWC, pH, CEC, OM, Ca^2+^, Na^+^, K^+^, C:N, TN and Mg^+^.

As with the peak greenness time points, we examined microbial communities from all sites at each time point using 16S- (Fig 3A) and lipid-based (Fig 3B) approaches and visualized community composition using NMDS. Both techniques yielded very similar patterns of community relatedness compared to the peak greenness data alone (Fig 2 vs. Fig 3). While including additional time points in the analysis increased the variability of communities at each site, communities still tended to group by sampling location rather than by time of sampling. In the 16S-based analysis, sampling site explained 72.3% (F = 69.003, p = 0.001) of community dissimilarity, while sampling time point (i.e., July, August, October, Feb/March) did not explain any significant portion of community dissimilarity (F = 1.003, p = 0.332). In lipid-based communities, sampling site explained 56.2% (F = 38.964, p = 0.001) of community dissimilarity; sampling time point also explained a small but significant portion of lipid-based community dissimilarity (3.5%, F = 7.381, p = 0.001). This result was also supported by pairwise and ANOVA analysis of NMDS scores, where some dates had distinct communities on one of the two NMDS axes, such as the July 10th sampling date in Florida. But for the most part, especially with regard to Utah soils, the sites clustered together regardless of the sample date (Table 2 and Fig 3).

**Table 2:** ANOVA and pairwise Tukey analysis to determine differences between sites and sample dates, based on PLFA and 16S analysis all dates and all sites (corresponds to Fig 3).

	16S				PLFA			
Site	MDS1	SD1	MDS2	SD2	MDS1	SD1	MDS2	SD2
AK 6/28/09	-0.075	0.077^**cd**^	-0.052	0.056^**abc**^	-0.073	0.051^**bc**^	-0.148	0.057^**fg**^
AK 8/8/09	-0.019	0.042^**bc**^	-0.099	0.034^**c**^	-0.140	0.023^**c**^	-0.219	0.073^**g**^
HI 7/2/09	-0.137	0.019^**d**^	0.021	0.062^**ab**^	-0.084	0.042^**bc**^	0.095	0.049^**ab**^
HI 8/13/09	-0.155	0.034^**d**^	0.029	0.055^**ab**^	-0.104	0.072^**bc**^	0.072	0.053^**abcd**^
HI 10/20/09	-0.159	0.033^**d**^	0.034	0.103^**ab**^	-0.074	0.053^**bc**^	0.147	0.083^**a**^
HI 2/11/10	-0.148	0.034^**d**^	-0.053	0.059^**bc**^	-0.129	0.030^**c**^	0.075	0.031^**abc**^
FL 7/10/09	0.005	0.031^**bc**^	0.045	0.042^**a**^	-0.082	0.1485^**bc**^	0.022	0.102^**bcde**^
FL 10/15/09	0.009	0.035^**b**^	0.038	0.025^**ab**^	-0.017	0.0542^**b**^	0.029	0.076^**bcde**^
FL 2/15/10	0.013	0.082^**b**^	0.041	0.083^**ab**^	0.020	0.0046^**b**^	0.075	0.039^**abcde**^
UT 7/15/09	0.251	0.049^**a**^	0.014	0.044^**ab**^	0.252	0.017^**a**^	-0.047	0.057^**ef**^
UT 10/3/09	0.231	0.041^**a**^	-0.032	0.036^**abc**^	0.253	0.017^**a**^	-0.030	0.058^**cde**^
UT 3/2/10	0.267	0.026^**a**^	0.023	0.050^**ab**^	0.254	0.020^**a**^	-0.035	0.026^**de**^
P-value	<0.0001		<0.0001		<0.0001		<0.0001	

Superscript letter values indicate statistical differences between clusters of communities associated with MDS1 (x-axis) and MDS2 (y-axis). SD indicates standard deviation.

We examined which environmental variables best explained the variation in 16S-based communities over time across all dates. Soil pH, SWC, CEC, Na^+^, Mg^+^, and Ca^2+^ ion concentrations explained significant portions of 16S-based community dissimilarity (Table 3). Similarly, we examined which environmental variables best explained lipid-based community dissimilarity and found that all measured environmental variables, with the exception of TC and SO_4_^−^ ion concentrations, explained a significant portion of community dissimilarity. SWC, pH, and CEC explained the highest proportions of dissimilarity, but soil OM, Ca^2+^, Na^+^, K^+^, C:N, TN, and Mg^+^ also explained smaller, but significant, portions of lipid-based community dissimilarity (Table 3). TMB and fungal:bacterial ratios were not included as explanatory variables for the lipid-based community because they are not independent of the response data. We performed stepwise multiple linear regression analysis to determine which factors best explained the variation in each NMDS axis. However, because a large number of environmental variables combined to explain small portions of overall community dissimilarity using both 16S- and lipid-based approaches, no one environmental variable explained a large portion of any NMDS axis.

**Table 3:** Factors explaining the variation in 16S-based and lipid-based communities across seasons at four ecosystem sites, as described by PERMANOVA analysis. NS indicates not significant.

	16S		PLFA	
Soil Properties	R^2^	p	R^2^	p
**pH**	**0.3246**	0.001	**0.1303**	0.001
**SWC**	**0.2185**	0.001	**0.2022**	0.001
**CEC**	**0.0869**	0.001	**0.1092**	0.001
**OM**	0.0475	0.001	0.0274	0.001
**Na** ^**+**^	0.0186	0.009	0.0499	0.001
**Mg** ^**+**^	0.0159	0.020	0.0147	0.013
**Ca** ^**2+**^	0.0131	0.041	0.0454	0.001
**K** ^**+**^	NS	NS	0.0311	0.001
**C:N**	NS	NS	0.0549	0.001
**TN**	NS	NS	0.0180	0.008

### Temporal Differences Within Sites

We examined the extent of 16S- and lipid-based microbial community variation over time at each site. 16S-based communities did not vary over the collection time points in soils from Alaska (ANOSIM R = 0.1735, p = 0.051), Hawaii (ANOSIM R = 0.09179, p = 0.094) or Utah (ANOSIM R = 0.05885, p = 0.164). However, 16S-based communities in Florida did vary temporally (ANOSIM R = 0.133, p = 0.013). Lipid-based communities did not change over time in Florida soils (ANOSIM R = 0.07567, p = 0.175) or Utah soils (ANOSIM R = 0.04049, p = 0.228), but did vary in soils collected from Alaska (ANOSIM R = 0.2065, p = 0.03) and Hawaii (ANOSIM R = 0.09045, p = 0.02).

**Fig 4:**
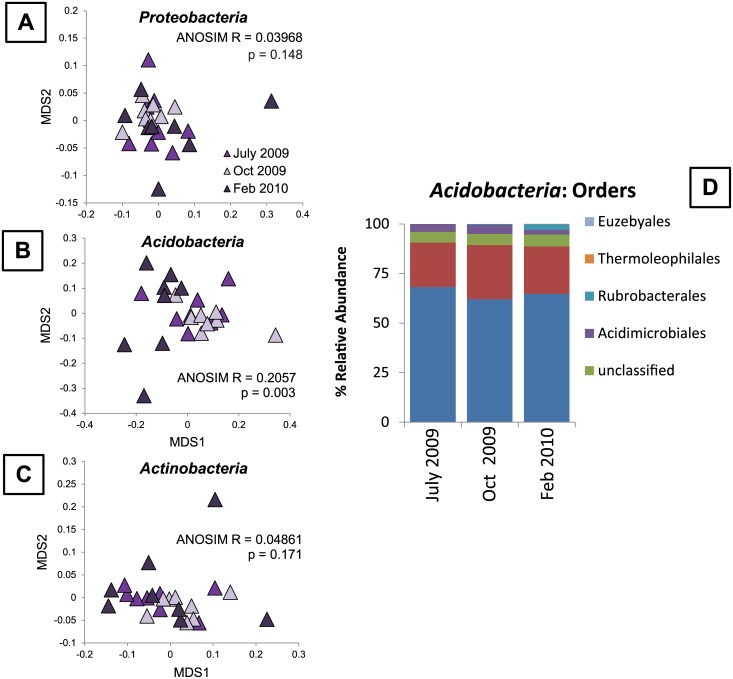
Changes in 16S rRNA-based community composition in Florida soils over time. (A) Relative abundances of all taxa classified at the phylum level and unclassified taxa over time. (B) NMDS ordination of all order-level taxa classified within the phylum *Proteobacteria*, which did not vary over time (ANOSIM R = 0.03968, p = 0.148). (C) NMDS ordination of all order-level taxa classified within the phylum *Actinobacteria*, which did not vary over time (ANOSIM R = 0.04861, p = 0.171). (D) NMDS ordination of all order level taxa classified within the phylum *Acidobacteria*, which varied significantly over time (ANOSIM R = 0.2057, p = 0.003).

We investigated the underlying factors driving changes in soil microbial community composition over time in the 16S-based communities from Florida soils and in the lipid-based communities from Alaska and Hawaii soils. In Florida, none of the relative proportions of the top phyla identified using 16S sequencing changed significantly over time (Fig 4A). We reclassified all sequences within each of the dominant phyla and examined shifts in within-phyla community composition at the order level. Within *Proteobacteria* and *Actinobacteria*, there were no shifts in order-level community composition over time (Fig 4B and 4C). However, within *Acidobacteria*, the order-level communities in Florida soils shifted significantly over time (Fig 4D). We tested whether any of the measured environmental variables explained the dissimilarity within *Acidobacteria* in Florida soils using a permanova; no variables were significant, though CEC explained 11.6% of the dissimilarity (p = 0.059). In Alaskan soils, all categorized lipids remained proportionally the same over time (Fig 1), with the exception of SF, which decreased significantly from June to August (F _1,13_ = 11.235, p = 0.005). Nonspecific lipids, or those that were not diagnostic for a particular bacterial or fungal group, increased over time (F_1,13_ = 7.609, p = 0.016). In Hawaiian soils, all the categorized and nonspecific lipids remained at the same proportion over time (Fig 1), with only the lipids diagnostic for GN increasing incrementally between October and February (F _3,23_ = 3.49, p = 0.032). These results suggest that, while 16S and lipid-based approaches provide similar results when comparing broad differences among dissimilar communities (i.e., across sites), they provide distinctly different information when examining how highly similar communities (i.e., within sites) vary over time.

### Effect of Soil Storage Temperature on 16S-Community Composition

**Fig 5:**
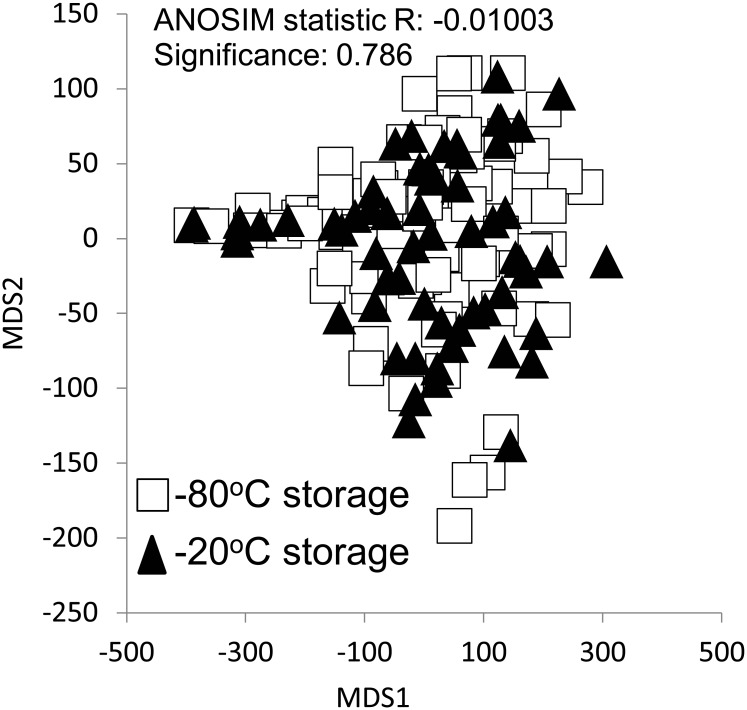
Comparison of the effect of soil storage temperature (-80°C vs. -20°C) on the 16S rRNA-based communities generated from soils collected from Hawaii. There was no significant effect of storage temperature on the communities examined at the phylum level (ANOSIM R = -0.01003, p = 0.786).

We examined the effect of the soil storage temperature on 16S rRNA gene-based community composition using soils collected from the Hawaiian site across five time points. Soils were stored for at least six months at either -80°C or -20°C prior to DNA extraction and analysis. 16S-communities did not differ by storage temperature (ANOSIM R = -0.01003, p = 0.786), indicating that the difference between -80°C and -20°C storage temperature has no effect on broad DNA-based indicators of soil microbial community composition (Fig 5).

## Discussion

### Differences Among Sites

Examining edaphic influences at peak plant productivity across four biomes results in three distinct groupings or variables that predominately influence microbial community composition: (1) tropical/subtropical moist broadleaf and taiga soils with high SWC and OM (Laupahoehoe, Hawaii and Caribou-Poker Creek, Alaska), (2) temperate, semiarid grassland/savanna/shrubland soils with more alkaline pH (Onaqui Benmore, Utah), and (3) tropical/sub-tropical coniferous forest soils with low CEC and cation concentrations (Ordway Swisher, Florida). We hypothesized that a few ecologically meaningful environmental filters could characterize the variation in soil microbial communities across broad environmental gradients. Our results corroborate other studies indicating that site-specific edaphic factors explain the majority of variation among soil microbial communities, and suggest that at broad-scales, inferences of microbial composition might be made from knowledge of soil biogeochemistry [16]. Exclusive bacterial 16S rRNA techniques and more inclusive lipid-based profiling techniques that include fungi, archaea, and other lipids that are non-bacterial, were both strongly driven by soil pH, SWC, and CEC. More specifically, soil pH explained the majority of community dissimilarity along MDS1 (Figs 2B and 3D, X-axis) using both analyses, while SWC explained the majority of community dissimilarity along MDS2 (Figs 2B and 3D, Y-axis) regardless of the technique used to examine microbial community composition. CEC, or concentrations of various measured cations (i.e., Ca^2+^, Na^+^, K^+^) explained some portion of the variation on both axes, indicating that CEC is a global variable that explains community dissimilarity along multiple vectors. A recent study of soil bacterial and fungal communities across soil and land-use types in France shows that pH, trophic resources, texture, and land use predominantly explain community composition [16]. In previous studies that examined 16S rRNA-based soil microbial community variation over nine biomes, pH was shown to be the single edaphic factor explaining community composition at the phylum level [11], [12], and no other environmental variables added to an explanation of community dissimilarity. In both our study and these previous studies, soil pH ranged from < 4 to > 8. However, previous studies measured soil moisture deficit instead of SWC, and did not measure CEC [11], [12], so these variables were not tested as explanatory factors driving soil microbial dissimilarity. Since soil moisture deficit is a potential measurement (i.e., the amount of water soil can hold against gravity) and not a field-level measurement of the actual water availability in soil at a particular time point, this could explain the discrepancy among studies and account for the high explanatory power that SWC exhibited in this study.

### Temporal Variation Within Sites

Seasonal and diel wetting and drying cycles are important natural disturbances to microbial communities that can have a significant impact on microbial diversity and function [48]. Low pH at a time point when SWC is low may result in a low CEC (i.e., tropical/sub-tropical coniferous forest, Florida), while soil with an inherently higher pH at a time point of low soil water content may have a high CEC (i.e., temperate grassland/savanna/shrubland, Utah). Combined changes in pH, SWC, and CEC could be linked to direct physiological constraints on soil microorganisms, and soils that exhibit frequent shifts in these local environmental variables may select for more resilient taxa that are physiologically able to tolerate stress caused by fluctuating conditions and disturbance [21]. As such, soils that exhibit the greatest variability in pH, SWC, and CEC may harbor soil microbial communities that are more resilient to future disturbances caused by climate change, while more stable soil environments may contain communities that are at higher risk of disrupted structure and function. Growing evidence from a range of studies and ecosystems suggests these overarching variables can be used to guide hypotheses of soil microbial diversity and hotspots of activity and serve as a metric by which to measure and predict change.

As the myriad of microbial community profiling techniques continues to grow with rapid advancement in sequencing technology, it is becoming possible to compare analyses of gene-independent microbial composition (i.e., DNA-based shotgun metagenome) and growth (i.e., lipid-based and RNA-based). DNA-based studies that examine temporal changes in soil microbial communities have demonstrated that they are fairly stable over time [1], [18], and that shifts in community composition are usually associated with a direct disturbance [12], [49] rather than time alone. For instance, soil exposed to heavy metals [50], enrichment of organic matter [51] or land use change [19], [52], all result in clear changes in the structure of soil microbial communities over time, whereas undisturbed communities typically remain stable [18], [53]. A meta-analysis of 16S rRNA gene sequencing across a range of environments revealed soil microbial communities to be among the least variable over time [18]. The authors suggest that longer-term seasonal and annual observations may reveal temporal changes in soil bacterial communities. In our 1-year survey, we still do not find strong temporal 16S rRNA-based patterns of community variability in three of the four ecosystems studied. As suggested in [18], high small-scale spatial heterogeneity may mask these community turnover signatures. For the purposes of future studies, these results provide a baseline of data to examine shifts in soil microbial communities though surveys spanning decades or more, which is a more relevant time scale for examining soil microbial community response to climate change.

### Differences Among Sites Over Time

When communities from all sites were examined over time, our results indicate that 16S rRNA-based community composition did not vary significantly over time, and that sample provenance was the main factor influencing dissimilarity among soil microbial communities. Lipid-based analysis also indicates that the majority of the variation in community composition was explained by sample provenance (56.2%), but that a small but significant percentage of variation was also explained by sample date (3.5%). Changes in lipid biomass indicate a direct response of soil microbial communities to plant growth and exudates over shorter timeframes, which provide key energy resources for soil microbial communities [54]. Plant exudates trigger a change in soil microbial communities, potentially leading to enhanced SOM mineralization, known as the priming effect [55], [56]. The variation in lipid-based community composition described here may reflect plant community-soil interactions as it relates to unique biomes. For example, in this study, the microbial biomass of the tropical broadleaf forest site (Hawaii) was over four times greater than that of the other ecosystems. This reflects the high plant productivity, and thus high levels of both labile carbon input into soils and of stable SOM, that is characteristic of tropical and subtropical ecosystems [57], [58]. A comparison of neotropical forests that differ in plant species diversity along a precipitation gradient showed microbial biomass (measured using the same PLFA technique) to be significantly correlated with mean annual precipitation but not with plant diversity [59].

When we examined each site individually, we observed some differences in the soil characteristics that explained the majority of between-site community variability (i.e., pH, SWC, CEC). In Alaska, pH increased from June to August; in Utah, pH increased in March above pH levels that were recorded in July and October; in Florida, pH increased in October and then returned to the pH recorded at peak greenness in February. SWC increased in October in both Florida and Utah above the levels recorded at peak greenness. Finally, CEC only changed in Florida soils, increasing incrementally over time. Of the four ecosystems, Florida was the only one that experienced shifts in soil pH, SWC, and CEC over time. Sites other than Hawaii, where variability was comparably low, experienced shifts in only 1–2 of these descriptive variables, but not all three of them.

When sites were examined independently, we only observed a significant shift in 16S rRNA-based community composition over time in the Florida soils, and none of the other sites exhibited a significant temporal shift. The cause of this shift might be attributed to the observation that taxa within *Proteobacteria* and *Actinobacteria* were relatively stable in Florida soils, but that taxa within *Acidobacteria* changed over time. *Acidobacteria* are ubiquitous members of soil microbial communities and high abundances of *Acidobacteria* tend to correlate with low soil pH [60], [61], and different subgroups of *Acidobacteria* dominate forested versus grassland soils [62]. However, within subgroups of *Acidobacteria*, other environmental factors (such as soil N) are related to shifts in community composition [62]. In our study, none of the measured environmental variables explained the observed shift in *Acidobacteria*, but CEC explained the greatest proportion of dissimilarity (11.6%). It is possible that greater replication at each time point would yield further explanatory power. However, our study, coupled with evidence from previous studies mentioned above, suggests that *Acidobacteria* subgroups are more useful indicators of fine-scale changes than the 16S rRNA-based bacterial community as a whole. As such, it may be useful to consider *Acidobacteria* as “sentinel microbial taxa” since they might act as early indicators of the effects of climate change within the soil environment.

Lipid-based community composition did not fully reflect our observations with DNA-based data. When we examined temporal variation within each site using lipid-based community analyses, we observed significant temporal changes in soils collected in taiga and tropical forests (Alaska and Hawaii), but not in the grassland/shrubland and conifer sites (Utah and Florida). The shift in lipid-based communities in Alaska correlated with a significant increase in soil pH and was accompanied by a decrease in the fungal:bacterial ratio from June to August, as well as an increase in SF lipids and nonspecific lipids. However, in Hawaii, none of the measured environmental variables changed over time, but TMB and GN bacterial lipids both decreased in October.

### Comparison of Analytical Techniques

While broad comparisons across distinct ecosystems and biomes yields similar results with DNA- and lipid-based approaches, the unique benefits of each technique are evident when examining highly similar communities over time. It is likely that DNA-based approaches for sampling bacterial diversity are more sensitive to changes in the soil abiotic microenvironment, namely pH, SWC, and CEC, as seen in Florida soils. DNA-based methods use small amounts of soil and examine tiny fractions of the microbial community in great detail, with high enough resolution to examine changes within specific bacterial taxonomic groups, such as *Acidobacteria*. Thus, in a subtropical conifer forest/grassland system in Florida with low variability in MAT but distinct rainfall patterns, only 16S rRNA community composition varied. However, lipid-based approaches appear more sensitive to microbial community changes that correlate with the soil biotic environment, and are more linked to the aboveground plant community. The Alaskan taiga, even in the short time period sampled in this study, experienced seasonal changes in temperature and soil moisture, which influenced plant phenological responses. These factors likely influenced the rapidly responding, metabolically active microbial community captured by our lipid-based methods, particularly soil fungi [63]. Similarly, the substantial plant community and microbial biomass levels in the Hawaiian tropical broadleaf forest likely influenced the active lipid-based community. For example, lipid-based community composition has been shown to respond drastically to invasive plant encroachment in this system, particularly affecting the relative abundance of saprophytic fungi [64]. Together these studies highlight the important role of fungal diversity in lipid-based community analyses, and the addition of fungal gene sequence data (i.e., ITS, 18S rRNA) could elucidate the fungal contribution to lipid-based data. Furthermore, the addition of RNA-sequence data might allow for a better nucleic-acid based estimate of the active microbiota [21] and hence a clearer comparison with the lipid-based data.

### Comparison of Soil Sample Storage

Given the diverse insights to be gained from combining a myriad of analyses, investments in soil sample archives that scale from microns across ecosystems will provide a powerful tool for understanding the mechanisms that govern microbial ecology, and the microbial role in sustainable ecosystem function. One challenge with sample archive is determining a feasible, consistent procedure that ensures sample integrity and allows for decadal comparisons. We examined the effect of storage temperature on tropical Hawaiian soils for at least six months prior to analysis and found that storage at -80°C versus -20°C does not impact 16S rRNA-based community structure. In a similar investigation, soil storage temperature (-20°C and -80°C) and time (14 days) did not alter 16S rRNA pyrosequence-based community composition [65]. However, using Illumina iTAGs, significant differences in community structure due to variation in storage time and temperature were observed [66], suggesting that the limited sampling depth from 454 pyrosequencing technology is insufficient to capture this change. Furthermore, the provenance of the sample may strongly influence its sensitivity to storage. For example, microbial communities from lowland tropical forests and tundra are acclimatized to different soil moisture and temperature regimes and it is reasonable to expect that cold storage will have significantly different effects. Furthermore, RNA and protein-based studies are even more sensitive than relatively recalcitrant DNA molecules and require even more stringent preservation conditions. This highlights a number of ongoing issues that could limit comparisons across studies and systems that can be partially alleviated by community-driven standardization of sample collection, preservation, and metadata information as promoted by the Genomics Standards Consortium.

### Conclusion

Free, open access datasets are powerful tools for integrating research and teaching in undergraduate and graduate student classrooms. They can be a useful resource for fostering interdisciplinary collaborations, testing the relevance of ecological theory and models, and developing novel hypotheses, particularly when communities of sentinel organisms such as microorganisms corroborate or contrast patterns, processes, and behaviors of macro-organisms. The combination of sequencing advances that characterize the taxonomic and physiological attributes of microscopic communities and the investment in ecological observatory infrastructure that incorporates whole-ecosystem monitoring will provide unprecedented spatial and temporal resolution for testing ecological hypotheses in soil microbial communities, for example: How is the structure of soil microbial communities generated and maintained? How stable is community composition and the relative abundance of taxa over space and time? What proportion of community variation is driven by dominant, common, or rare taxa and how do these abundance distinctions correlate with microbial functional groups? How resilient are microbial communities to punctuated disturbance or gradual environmental change? The accumulation of these extensive datasets allows not only monitoring, but provides a powerful resource of baseline data from which new hypotheses about the response of community and ecosystem change to disturbance events can be developed. These resources will be especially useful to early career investigators who may be limited in their ability to generate preliminary data. It is therefore critical that current students across disciplines gain exposure to use and interpretation of these data streams.

## Supporting Information

S1 Table NEON metadata, including PLFA data, for SRA study SPRP061236. These metadata follow the guidelines of the Genomics Standards Consortium (GSC) minimum information about a marker gene sequence (MIMARKS) [29] and also include PLFA data for each sample.
(XLSX)
